# Combined Protective Effects of Raffinose and Isoflavones Against High-Fructose-Induced Liver Damage via Improved Bioavailability and Metabolic Regulation

**DOI:** 10.3390/foods15173094

**Published:** 2026-08-31

**Authors:** Yingmei Wu, Xiangnan Zhang, Yu Wang, Ting Li, Yalong Lu, Xingbin Yang

**Affiliations:** 1 Shaanxi Engineering Laboratory for Food Green Processing and Safety Control, and Shaanxi Key Laboratory for Hazard Factors Assessment in Processing and Storage of Agricultural Products, Engineering Research Center of High-Valued Utilization of Fruit Resources in Western China, Ministry of Education, College of Food Engineering and Nutritional Science, Shaanxi Normal University, Xi’an 710119, Chinaluyalong@snnu.edu.cn (Y.L.); 2 College of Biology and Food Engineering, Chongqing Sanxia University of Science and Technology, Chongqing 404120, China

**Keywords:** soybean isoflavones, raffinose, absorption, dyslipidemia, liver protection

## Abstract

This study investigated the combined administration of raffinose (RAF) and isoflavones (ISO) to enhance ISO bioavailability and alleviate high-fructose (HF)-induced liver damage. Mice were administered 20% HF water with RAF, ISO, or their combination (RAF + ISO) for 10 weeks. UPLC-qTOP/MS analysis showed that RAF + ISO significantly increased total ISO levels in serum, urine, and feces by 18.5%, 37.4%, and 13.6%, respectively, compared to ISO alone (*p* < 0.05). The combined treatment more effectively reduced HF-induced weight gain, visceral fat accumulation, and dyslipidemia, partly through suppression of FAS and ACC gene expression (*p* < 0.05). Furthermore, RAF + ISO more efficiently attenuated hepatic oxidative stress, inflammation, and insulin resistance than either agent alone. Liver enzyme activities (AST, ALT, ALP) were significantly reduced by RAF + ISO treatment, consistent with histopathological improvements in liver tissue. These findings demonstrate that RAF enhances the systemic availability of ISO and potentiates its protective effects against HF-induced metabolic and hepatic alterations.

## 1. Introduction

High fructose (HF) consumption serves as a primary contributor to nonalcoholic fatty liver disease development due to its aggravative effect on hyperglycemia and adipogenesis [1,2]. Our previous studies pointed out that excessive fructose consumption resulted in dyslipidemia, endothelial dysfunction, oxidative stress, insulin resistance, adiposity and hepatotoxicity in mice [3]. Despite growing awareness of the role of excessive fructose in aggravating liver injury and disrupting lipid metabolism, the development of suitable preventive and treatment strategies remains limited. Therefore, the available preventive method for HF-induced hepatic injury and lipid metabolic disorder needs to be established urgently. Fortunately, numerous studies have identified promising preventive and therapeutic roles of dietary interventions in mitigating various fatty liver diseases associated with impaired glucose and lipid metabolism [4,5].

It is well known that multiple healthy effects of soybean foods are attributed to their nutrient-enriched carbohydrates, proteins, isoflavones and minerals [6,7]. Soybean as the richest source of isoflavones (ISO), exerts protective effects against many chronic diseases related to it because of its antioxidant, anti-inflammatory and hypolipidemic regulation properties [8,9]. Despite its health-promoting properties, ISO exhibits low oral bioavailability, mainly due to limited aqueous solubility and poor intestinal permeability [10]. Emerging studies suggest that certain non-digestible functional sugars can facilitate flavonoid uptake in the gut. Previous studies from our group have demonstrated that nondigestible carbohydrates can modulate the bioavailability and biological effects of soybean isoflavones. Stachyose enhanced isoflavone bioavailability and ameliorated hyperlipidemia and hyperglycemia in high-fat-diet-fed mice [11,12,13], and co-administration of stachyose with genistein enhanced its hepatoprotective effects in mice subjected to chronic high-fructose consumption [13,14,15]. Similarly, soybean soluble polysaccharides have been shown to increase genistein availability and potentiate its biological effects [16,17]. These findings provide evidence that nondigestible carbohydrates can interact with soybean isoflavones and influence their in vivo efficacy.

However, whether raffinose, a structurally distinct nondigestible oligosaccharide naturally occurring in soybeans and other plant foods, can enhance the systemic availability of soybean isoflavones and potentiate their protective effects against high-fructose-induced metabolic and hepatic alterations remains unclear. Therefore, the present study investigated the effects of RAF, ISO, and their combination in HF-fed mice and evaluated ISO availability using UPLC-qTOF/MS. To the best of our knowledge, this is the first study demonstrating that raffinose specifically enhances the systemic availability of soybean isoflavones and potentiates their protective effects against high-fructose-induced metabolic and hepatic alterations.

## 2. Materials and Methods

### 2.1. Chemicals and Reagents

Raffinose (≥98% purity) and soybean isoflavones (≥95%) for animal studies, together with analytical standards of daidzein, glycitein, and genistein (all ≥98%), were supplied by Shanghai Yuanye Bio-Technology (Shanghai, China). Kits for total cholesterol (TC), triglycerides (TG), HDL-C, LDL-C, ALT, AST, and ALP were sourced from Changchun Huili Biotechnology (Changchun, China). Assay kits for T-SOD, GSH-Px, MDA, and hydroxyl radicals, and ELISA kits for FFA, Nrf-2, insulin, glucose, IL-1, IL-6, TNF-α, TGF-β1, and leptin were purchased from Nanjing Jiancheng Bioengineering Institute (Nanjing, China). Primary antibodies against FAS, ACC, and GAPDH came from Proteintech (Rosemont, IL, USA); HRP-conjugated secondary antibodies from Santa Cruz Biotechnology (Dallas, TX, USA); and ECL reagents from Thermo Scientific (Rockford, IL, USA). All other chemicals were of analytical grade.

### 2.2. Animals and Experimental Design

Forty 4-week-old male Kunming mice (Air Force Medical University Animal Center) were housed at 22 ± 2 °C, 55–65% humidity, on a 12 h light/dark cycle, with chow and water ad libitum. No formal a priori power calculation was performed. The sample size (*n* = 8 per group) was determined based on previous studies from our group using comparable mouse models and experimental endpoints [18], in which similar group sizes were sufficient to detect biologically meaningful differences, while also considering the principle of minimizing the number of animals used. After a 1-week acclimation, mice were assigned to five experimental groups (*n* = 8 per group) using a random-number-based method, with body weight considered to ensure comparable baseline body weights among groups. Potential confounding factors were minimized by using mice of the same sex and similar age and body weight and by maintaining identical housing and environmental conditions across groups. The groups were as follows: Normal control (ND); high-fructose (HF; 20% fructose in drinking water); HF + raffinose (300 mg kg^−1^ day^−1^; HF/RAF); HF + isoflavones (300 mg kg^−1^ day^−1^; HF/ISO); and HF + raffinose + isoflavones (each at 300 mg kg^−1^ day^−1^; HF/RAF + ISO). Treatments were administered once daily by gavage for 10 weeks. All procedures followed Air Force Medical University guidelines (Approval No. 32072175).

### 2.3. Assay for Glucose Homeostasis

The oral glucose tolerance test (OGTT) was performed according to a previously published method with minor modifications [18]. After a 12 h fasting period, mice were orally administered glucose at a dose of 2 g kg^−1^ body weight. Blood glucose concentrations were measured using a commercial glucometer before glucose administration (0 min) and at the indicated time points after glucose loading. Fasting serum insulin levels were determined using a commercial ELISA kit according to the manufacturer’s instructions. Insulin resistance was estimated using the homeostatic model assessment of insulin resistance (HOMA-IR), calculated as follows: HOMA-IR = [fasting glucose (mmol L^−1^) × fasting insulin (mU L^−1^)]/22.5.

### 2.4. UPLC-qTOF-MS Quantification of Isoflavones

The quantification of isoflavones by UPLC-qTOF-MS follows our previous method [19]. The limits of detection (LOD) and quantification (LOQ) were determined at signal-to-noise ratios (S/N) of approximately 3 and 10, respectively. The LOD/LOQ values were 0.1/3.0 ng mL^−1^ for daidzein, 0.1/2.0 ng mL^−1^ for glycitein, and 50.0/250.0 ng mL^−1^ for genistein. Serum or urine (200 µL) was mixed with 1 mL methanol, centrifuged, filtered (0.22 µm PVDF), and analyzed. Feces (1.5 g) were homogenized in saline (1:6, *w*/*v*), centrifuged, and the supernatant (100 µL) was processed identically. An UltiMate 3000 UPLC (Thermo Fisher) equipped with a 2.2 µm C18 column (3.0 × 100 mm) was operated at 25 °C. Elution (0.2 mL min^−1^) used 40 mM ammonium formate (A) and acetonitrile (B): 0–10 min, 95 → 10% A; 10–12 min, 10% A; 12–15 min, 10 → 95% A; 15–20 min, 95% A. An ESI source scanned 50–1000 *m*/*z* or selected ions (SIM) for daidzein (*m*/*z* 253.2^−^), genistein (*m*/*z* 268.9^−^), and glycitein (*m*/*z* 283.2^−^).

### 2.5. Biochemical Assessments

For hepatic biochemical analyses, liver tissues were homogenized in ice-cold physiological saline at a ratio of 1:9 (*w*/*v*) and centrifuged, and the resulting supernatants were used for subsequent assays. Hepatic malondialdehyde (MDA), total superoxide dismutase (T-SOD), glutathione peroxidase (GSH-Px), hydroxyl radical scavenging activity, free fatty acids (FFA), Nrf-2, TNF-α, IL-6, IL-1, and TGF-β1 were measured using the corresponding commercial assay or ELISA kits (Nanjing Jiancheng Bioengineering Institute) strictly according to the manufacturer’s protocols. Absorbance was measured at the wavelength specified for each assay using a microplate reader. For quantitative assays, calibration curves were constructed using the standards supplied with the respective kits, and sample concentrations were calculated from the corresponding standard curves. Protein concentrations of liver homogenates were determined using the bicinchoninic acid (BCA) assay, and the relevant hepatic biochemical parameters were normalized to protein content where appropriate.

### 2.6. Western Blot Analysis

Liver lysates were prepared in ice-cold buffer containing PMSF and NaF, clarified (15,000 g, 15 min, 4 °C), and denatured with loading buffer. After SDS-PAGE and PVDF transfer, membranes were blocked (5% milk, 2 h, RT), probed with primary antibodies (1:1000, 4 °C overnight), then HRP-secondary antibodies (1:2000, 2 h, RT). Bands were visualized by ECL using a ChemiDoc-It 510 imaging system (UVP, Upland, CA, USA) and quantified using Image-Pro Plus 6.0 software (Media Cybernetics, Inc., Rockville, MD, USA).

### 2.7. Histology

Formalin-fixed (10%) liver (5 µm) and adipose sections were paraffin-embedded. Liver slides were stained with H&E or Oil Red O; adipose with H&E. Images were captured using an Olympus light microscope. Hepatic vacuolation was quantified in ImageJ 1.48; adipocyte area with Nano Measurer 1.2. Samples were coded before histological evaluation and quantitative analysis, and the investigators performing these assessments were blinded to the treatment-group allocation until the analyses were completed.

### 2.8. Statistical Analysis

Data are expressed as means ± SDs. Normality and homogeneity of variances were assessed prior to statistical analysis using the Shapiro–Wilk test and Levene’s test, respectively. Except for the oral glucose tolerance test (OGTT), differences among groups were analyzed using one-way analysis of variance (ANOVA), followed by Duncan’s multiple-range test for multiple comparisons. OGTT data were analyzed using two-way repeated-measures ANOVA, with treatment as the between-subject factor and time as the within-subject factor, including evaluation of the treatment × time interaction. Where appropriate, post hoc multiple comparisons were performed to compare treatment groups at individual time points. Statistical analyses were performed using GraphPad Prism 8, and differences were considered statistically significant at *p* < 0.05.

## 3. Results and Discussion

### 3.1. RAF Alters the Intestinal Fate and Enhances the Systemic Availability of ISO in HF-Fed Mice

**Figure 1:**
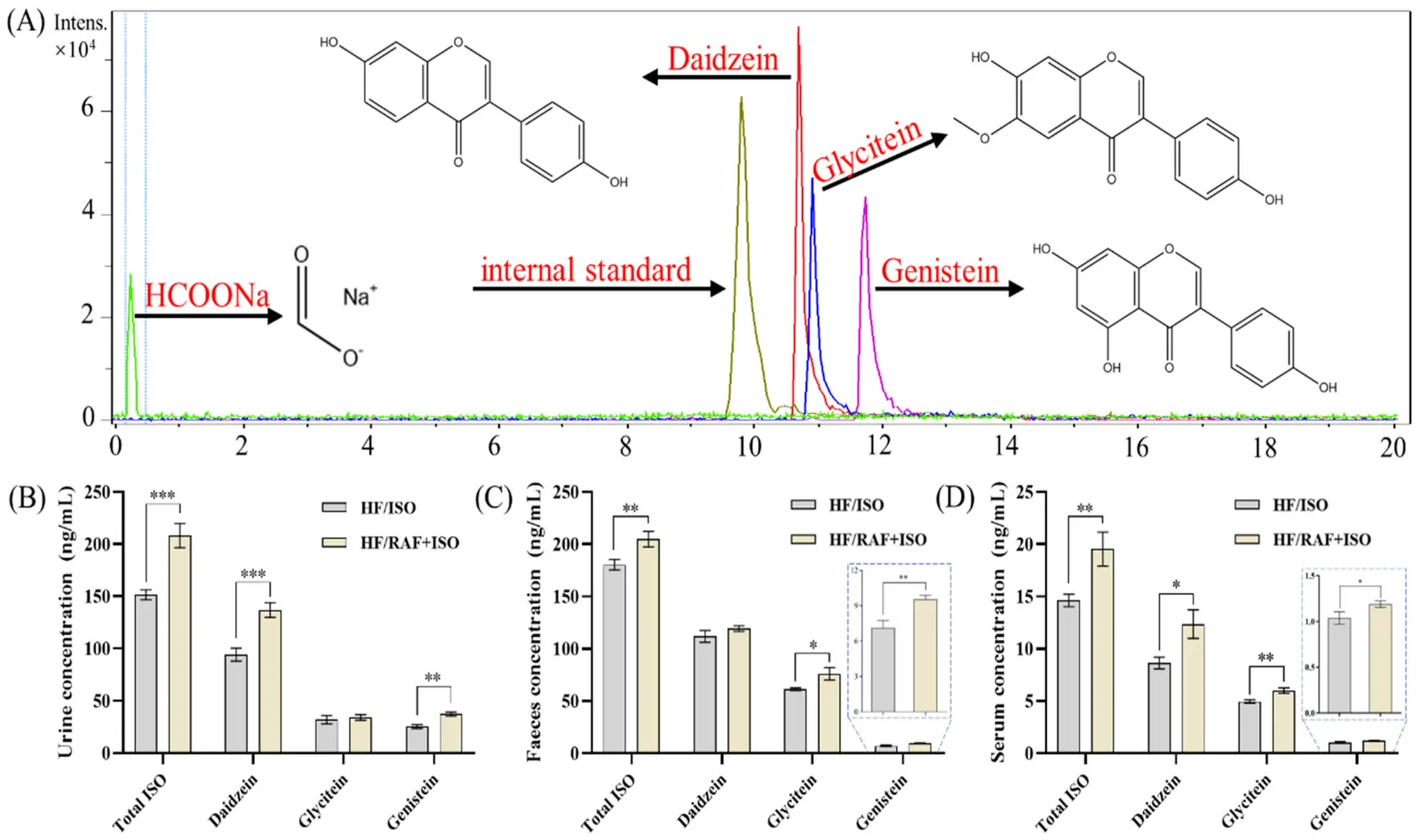
UPLC-qTOF/MS chromatograms of a standard substance mixture and chemical structures of daidzein, glycitein, and genistein (**A**), and urinary (**B**), fecal (**C**), and serum (**D**) concentrations of total isoflavones, daidzein, glycitein, and genistein in high-fructose (HF)-fed mice supplemented with isoflavones (HF/ISO) or co-supplemented with raffinose and isoflavones (HF/RAF + ISO). RAF, raffinose; ISO, soybean isoflavones; UPLC-qTOF/MS, ultra-performance liquid chromatography coupled with quadrupole time-of-flight mass spectrometry. Values are expressed as the mean ± SD. * *p* < 0.05, ** *p* < 0.01, and *** *p* < 0.001 compared with the ISO group.

As depicted in Figure 1A, the MRM chromatograms confirmed the identification of daidzein, glycitein, and genistein standards. Figure 1B–D show the concentrations of ISO in the urine, feces, and serum of ISO-treated and RAF + ISO-treated mice. Compared with ISO treatment alone, RAF + ISO co-treatment significantly increased the urinary concentrations of total ISO, daidzein, and genistein (*p* < 0.01; Figure 1B). Total fecal ISO levels were also significantly increased in the RAF + ISO group (*p* < 0.01; Figure 1C), whereas no significant difference was observed in fecal daidzein levels (*p* > 0.05). Importantly, co-administration of RAF and ISO significantly increased the serum concentrations of daidzein, glycitein, and genistein compared with ISO treatment alone (*p* < 0.05; Figure 1D).

The increased fecal ISO levels should not be interpreted as direct evidence of enhanced intestinal absorption. Rather, they suggest that RAF co-administration may alter the intestinal fate of ISO, potentially through changes in intestinal stability, microbial degradation, and/or intestinal metabolism. Previous studies have shown that nondigestible carbohydrates can influence the gastrointestinal disposition and bioavailability of flavonoids [18,20]. In contrast to the fecal findings, the significantly increased serum concentrations of daidzein, glycitein, and genistein provide more direct evidence that RAF co-administration enhances the systemic availability of ISO. The increased urinary excretion of ISO may also be consistent with increased systemic exposure. However, because intestinal permeability, transport, and uptake were not directly assessed in the present study, the specific mechanisms responsible for the altered disposition and increased systemic availability of ISO remain to be elucidated.

### 3.2. RAF + ISO Co-Treatment Alleviated Weight Gain and Adipocyte Enlargement

Chronic or excessive fructose intake is widely recognized as a major contributor to increased adiposity, lipid metabolic dysfunction, insulin resistance, and oxidative liver damage [3,6]. As shown in Table 1, water consumption remained comparable across all experimental groups (*p* > 0.05). However, mice consuming high-fructose (HF) water exhibited lower food intake than normal diet (ND) controls, likely due to the additional caloric load from fructose supplementation. As expected, the HF group showed the greatest overall body weight gain among the five cohorts. While final body weights among treatment groups (RAF, ISO, RAF + ISO) were not significantly different, animals receiving the combined RAF + ISO intervention displayed the lowest weight gain over time. Moreover, co-treatment significantly decreased hepatic and abdominal fat masses, accompanied by notable reductions in hepatic index and abdominal fat ratio compared to either agent alone (*p* < 0.05, Table 1), echoing previous reports [8,21].

**Figure 2:**
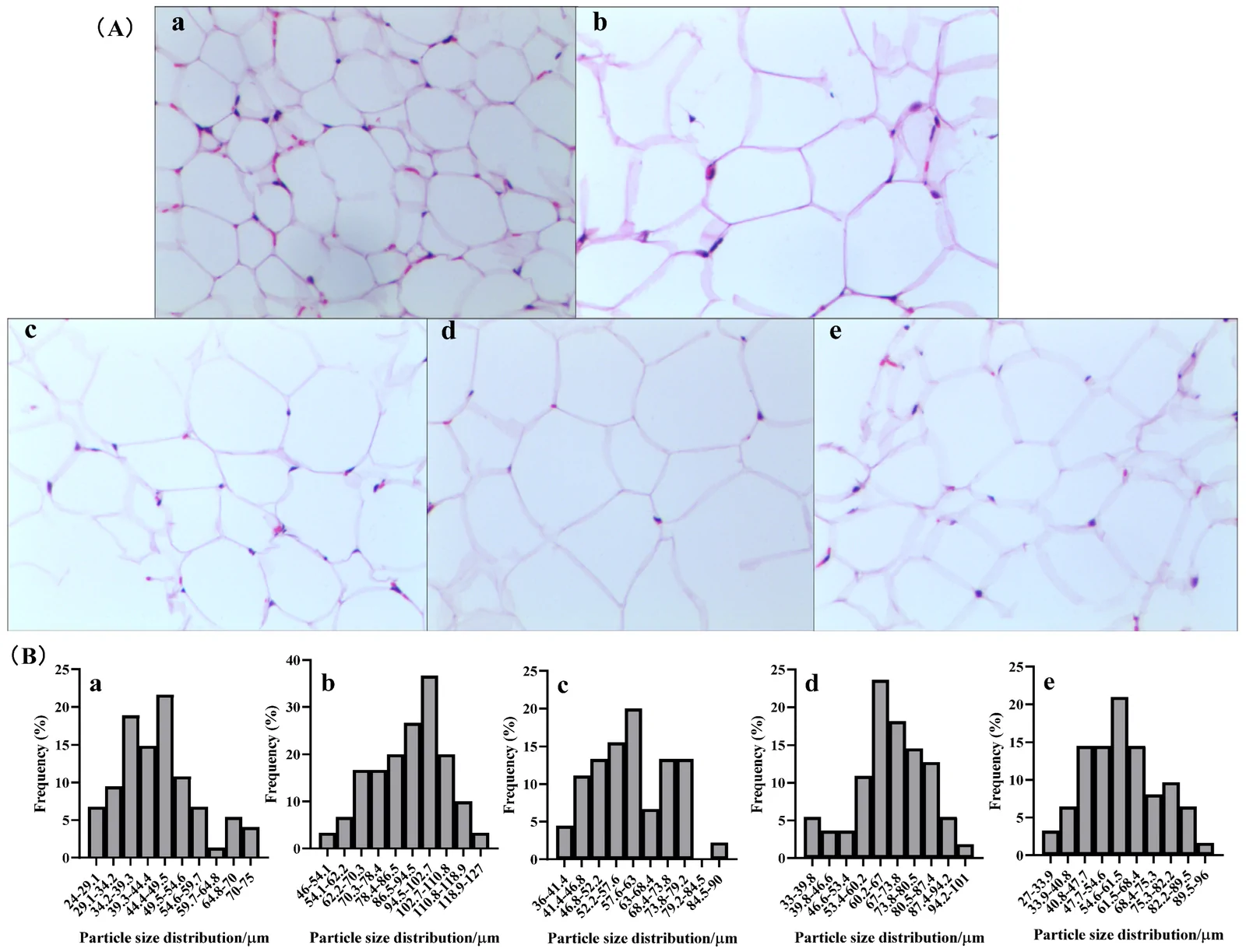
Hematoxylin and eosin (H&E) staining of adipose tissue (**A**) and size distributions of adipocytes (**B**) in the different experimental groups (a: Normal; b: HF; c: HF/RAF; d: HF/ISO; e: HF/RAF + ISO). HF, high fructose; RAF, raffinose; ISO, soybean isoflavones. Arrows indicate representative enlarged adipocytes. More than 500 cells in each group were counted (*n* ≥ 3).

To further explore adipose tissue alterations, we assessed adipocyte size and distribution using H&E-stained abdominal fat sections (Figure 2A,B). HF-fed mice exhibited enlarged adipocytes with a skewed distribution toward larger sizes, in line with increased fat deposition. Treatment with either RAF or ISO attenuated this hypertrophy, whereas their combination yielded a more substantial reduction in adipocyte size and normalization of distribution. These results indicate that RAF may potentiate ISO’s anti-adipogenic effects, contributing to improved regulation of body weight and fat storage.

### 3.3. RAF + ISO Co-Treatment Improved Glucose Tolerance and Lipid Profile in HF-Fed Mice

**Figure 3:**
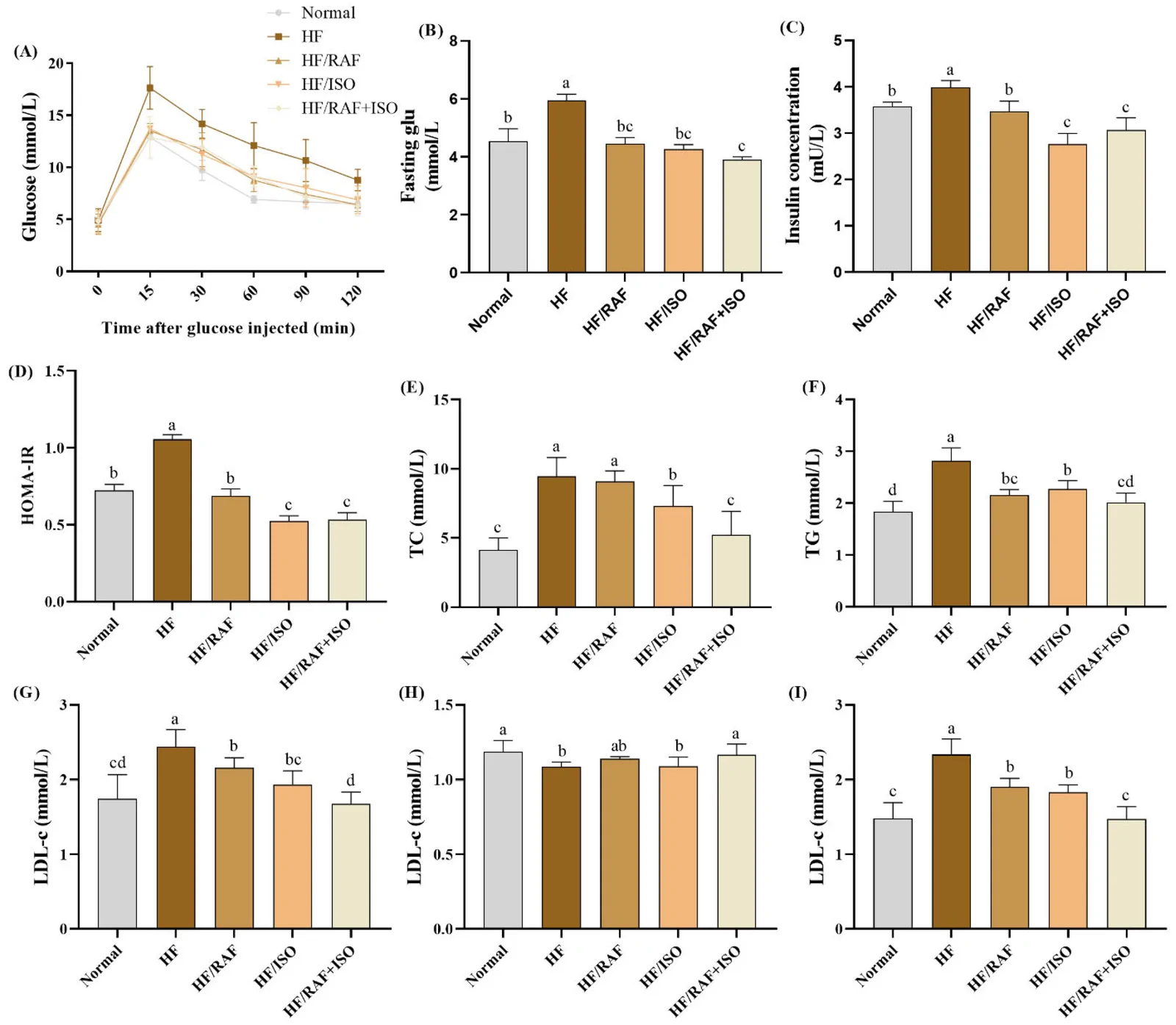
Oral glucose tolerance test (OGTT) (**A**), fasting glucose level (**B**), insulin concentration (**C**), homeostatic model assessment of insulin resistance (HOMA-IR) (**D**), total cholesterol (TC) (**E**), triglycerides (TG) (**F**), low-density lipoprotein cholesterol (LDL-C) (**G**), high-density lipoprotein cholesterol (HDL-C) (**H**), and LDL-C/HDL-C ratio (**I**) in the different experimental groups. HF, high fructose; RAF, raffinose; ISO, soybean isoflavones. Values are expressed as the mean ± SD (*n* = 8). Different letters indicate significant differences among groups (*p* < 0.05).

As shown in Figure 3A, oral glucose tolerance tests revealed a peak in blood glucose levels at 15 min post-administration, followed by a gradual decline. Mice on the HF diet exhibited significantly elevated glucose concentrations throughout the test period. Notably, co-administration of RAF and ISO produced a greater hypoglycemic effect than either treatment alone. After 10 weeks of HF exposure, fasting glucose in mice was increased by approximately 1.31-fold relative to controls (*p* < 0.05, Figure 3B). Treatment with RAF, ISO, and their combination led to substantial decreases in fasting glucose by 25.2%, 28.2%, and 34.4%, respectively. In parallel, serum insulin concentrations were significantly reduced in all treatment groups, with the greatest decline observed in the RAF + ISO group (*p* < 0.05, Figure 3C). The insulin resistance index (HOMA-IR) was markedly elevated in the HF group but significantly lowered following combination therapy (*p* < 0.05, Figure 3D). These findings suggest that RAF enhances the glucose-regulatory properties of ISO, contributing to improved glycemic control in HF-induced metabolic dysregulation.

Furthermore, insulin resistance is commonly linked with dysregulated lipid metabolism, and high fructose intake has been shown to provoke hyperlipidemia and hepatic lipid accumulation [18,22,23,24]. Consistent with previous studies [3,24], HF feeding significantly elevated serum levels of TG, TC, LDL-C, and the LDL-C/HDL-C ratio, while HDL-C was notably reduced (*p* < 0.05, Figure 3E–H). Both RAF and ISO treatments individually mitigated these disturbances. Importantly, the combined intervention achieved a more pronounced correction in lipid parameters, especially in lowering TC, TG, and the LDL-C/HDL-C ratio (*p* < 0.05, Figure 3I). This enhanced efficacy may stem from RAF-mediated improvement in ISO absorption, which likely boosts the active concentration of ISO in circulation and strengthens its modulatory effects on lipid metabolism [25,26]. Though small amounts of fructose (1–3%) can be converted into triglycerides, their contribution to lipogenesis is generally limited [27].

The gastrointestinal fate of RAF should also be considered when interpreting the enhanced effects of the combined intervention. As a nondigestible oligosaccharide, RAF is resistant to digestion in the upper gastrointestinal tract and reaches the colon, where it can be utilized by intestinal microorganisms. Although the gastrointestinal degradation and colonic fermentation of the RAF + ISO combination were not directly investigated in the present study, two complementary mechanisms may contribute to its biological effects. First, RAF co-administration significantly increased the serum concentrations of daidzein, glycitein, and genistein, indicating enhanced systemic bioavailability of ISO. RAF may influence the intestinal stability, metabolism, and availability of ISO, although the underlying processes remain to be elucidated. Second, colonic fermentation of RAF may modulate the intestinal microbial environment and generate microbial metabolites that exert additional metabolic effects. This possibility is supported by the improvements observed with RAF treatment alone in several metabolic, oxidative, and inflammatory parameters. Therefore, the greater efficacy of RAF + ISO may reflect both enhanced ISO bioavailability and the independent prebiotic and metabolic effects of RAF. Further studies evaluating gastrointestinal digestion, colonic fermentation, gut microbiota composition, and microbial metabolites are needed to clarify the mechanisms underlying the interaction between RAF and ISO.

### 3.4. RAF + ISO Combination Suppressed Lipogenesis and Reduced Fat Accumulation in HF-Fed Mice

**Figure 4:**
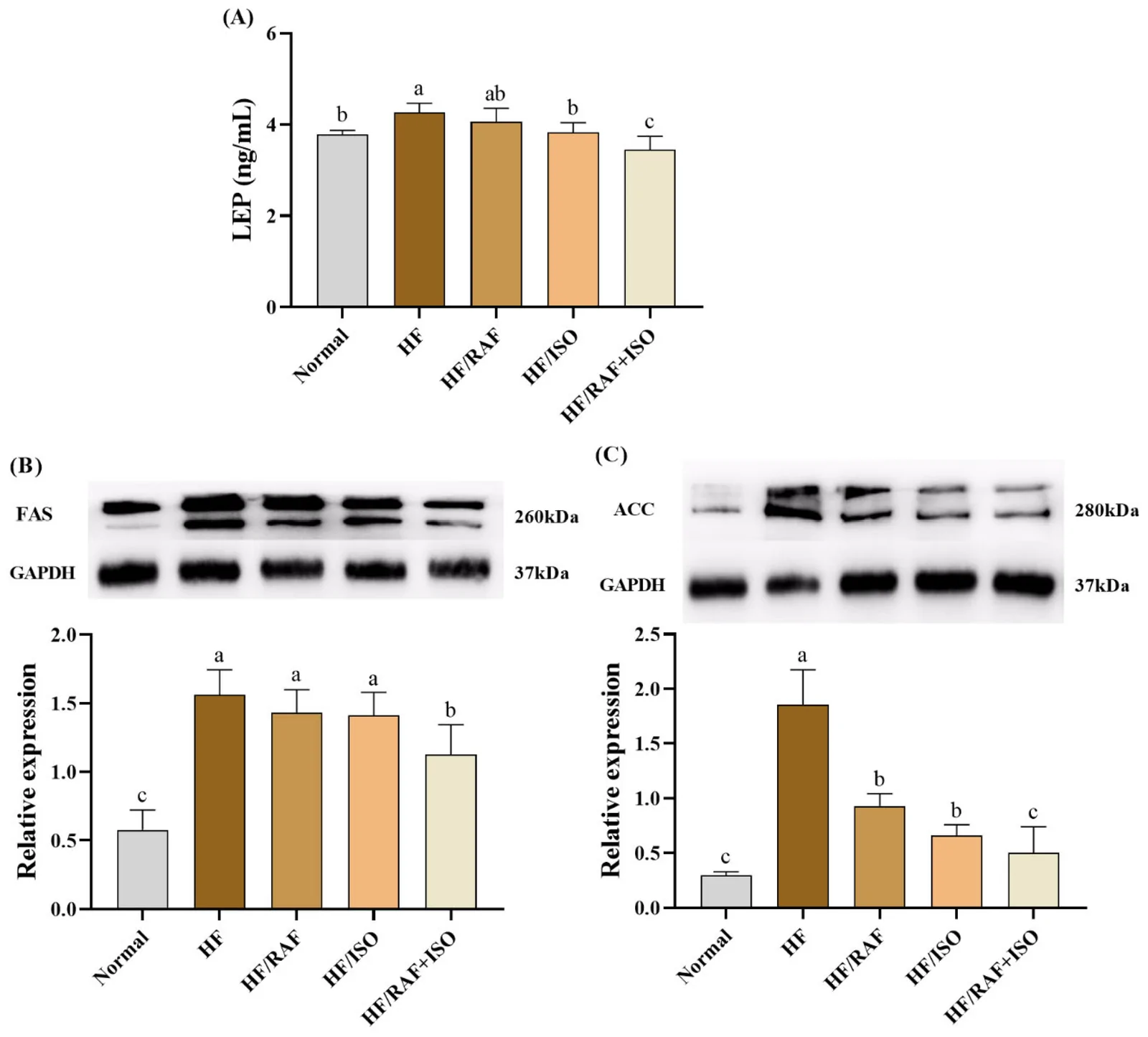
Effects of raffinose (RAF) and/or soybean isoflavones (ISO) on serum leptin (LEP) level (**A**) and the relative protein expression of fatty acid synthase (FAS) (**B**) and acetyl-CoA carboxylase (ACC) (**C**) in high-fructose (HF)-fed mice. Values are expressed as the mean ± SD (*n* = 8). Different letters indicate significant differences among groups (*p* < 0.05).

Leptin (LEP), a hormone closely linked to adiposity, is known to contribute to the development of obesity [18,27]. As illustrated in Figure 4A, mice receiving HF water exhibited a 1.25-fold increase in serum LEP levels compared to the normal control group (*p* < 0.05). Although administration of RAF or ISO alone partially reduced LEP levels, the co-administration of both agents resulted in a significantly greater decrease. This suggests that the combined intervention may be more effective in modulating fat-derived hormonal signals and controlling adiposity.

In addition, high fructose intake for 10 weeks markedly elevated the expression of fatty acid synthase (FAS) by 2.70-fold compared to the normal group (*p* < 0.05, Figure 4B). These data indicate that RAF + ISO may exert anti-obesity effects not only by modulating leptin secretion but also by interfering with lipogenic pathways. Previous findings have shown that ISO may suppress the activity of key transcription factors like ChREBP and SREBP-1c, which regulate genes such as FAS and ACC involved in de novo fatty acid synthesis [28]. As shown in Figure 4C, neither RAF nor ISO monotherapy was sufficient to significantly lower FAS expression. However, the combined treatment led to a pronounced downregulation of both FAS and acetyl-CoA carboxylase (ACC) levels. Specifically, ACC expression, which was 6.24-fold higher in HF-fed mice than in controls, was reduced by 3.71-fold following RAF + ISO administration (*p* < 0.05), a stronger inhibition than that achieved by RAF (1.99-fold) or ISO alone (2.81-fold).

The downregulation of FAS and ACC was consistent with observed decreases in serum triglycerides, total cholesterol, and insulin, supporting the hypothesis that co-treatment impairs lipogenesis. Since FAS and ACC are essential enzymes in the synthesis of fatty acids and triglycerides [20,29], their suppression likely contributes to the observed reduction in lipid storage and body fat accumulation.

### 3.5. RAF + ISO Combination Attenuated Oxidative Stress Induced by HF Consumption

**Figure 5:**
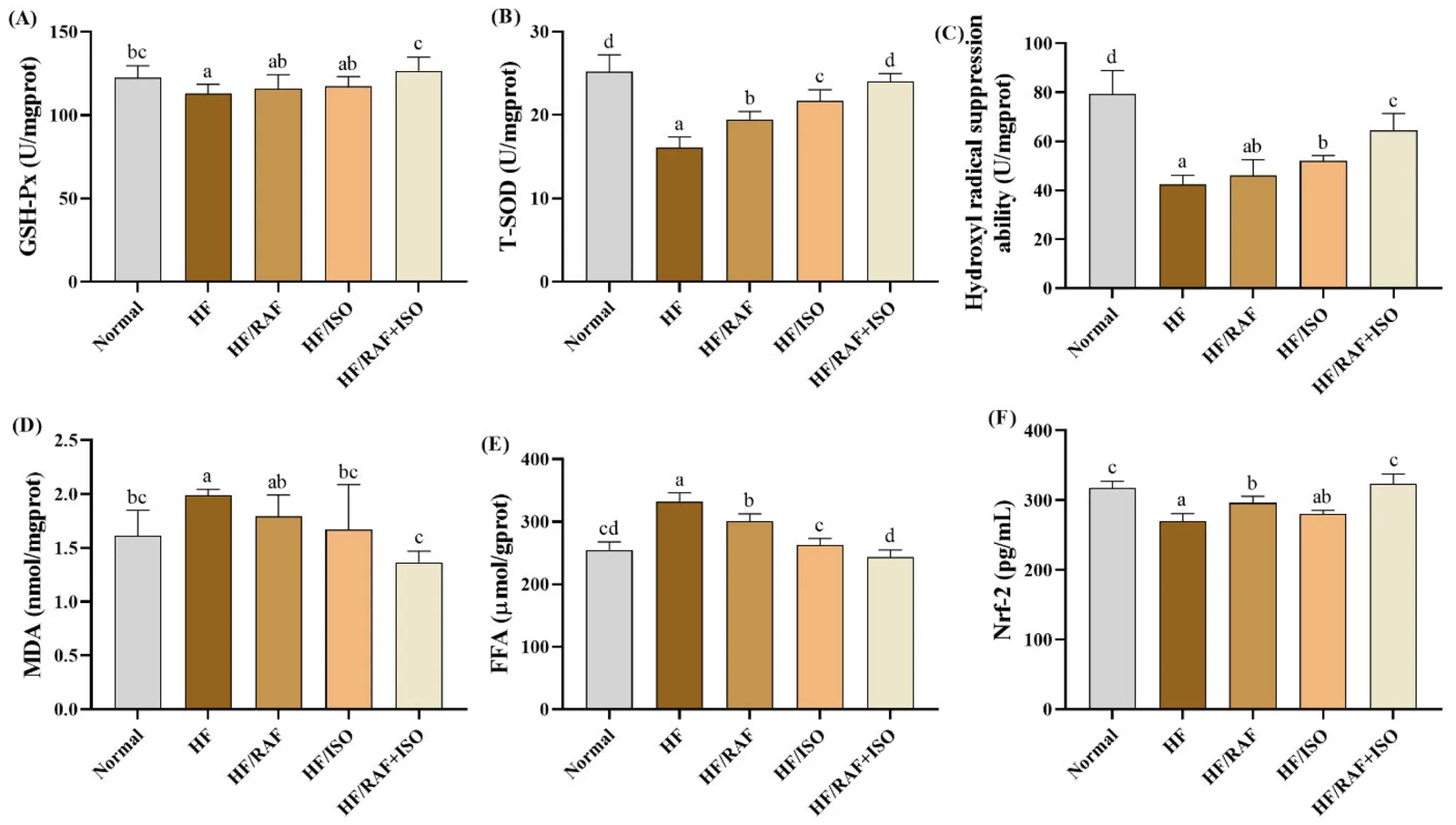
Effects of raffinose (RAF) and/or soybean isoflavones (ISO) on hepatic oxidative stress in high-fructose (HF)-fed mice. Hepatic glutathione peroxidase (GSH-Px) activity (**A**), total superoxide dismutase (T-SOD) activity (**B**), hydroxyl radical scavenging activity (HRSA) (**C**), malondialdehyde (MDA) level (**D**), free fatty acid (FFA) level (**E**), and nuclear factor erythroid 2-related factor 2 (Nrf-2) level (**F**) are shown. Values are expressed as the mean ± SD. Different letters indicate significant differences among groups (*p* < 0.05).

Hepatic accumulation of triglycerides is a well-known trigger of oxidative stress and lipid peroxidation, both of which contribute significantly to the onset and progression of nonalcoholic liver injury [30]. Malondialdehyde (MDA), a lipid peroxidation byproduct, has been widely recognized as a marker of oxidative damage to cellular membranes [3]. In addition, elevated concentrations of free fatty acids (FFA), especially those considered lipotoxic, have been implicated in the pathology of nonalcoholic fatty liver disease (NAFLD) [31,32]. As shown in Figure 5A–C, mice fed a high-fructose diet exhibited significant reductions in hepatic levels of GSH-Px, T-SOD, and hydroxyl radical scavenging capacity (HRSA), reflecting compromised antioxidant defense (*p* < 0.05). Treatment with either RAF or ISO individually improved these antioxidant parameters, while their combined administration produced more marked restoration of antioxidant enzyme activity (*p* < 0.05). Concurrently, RAF + ISO co-treatment significantly lowered hepatic MDA content by 31.3% relative to HF-fed controls (*p* < 0.05), suggesting mitigation of lipid peroxidation.

Furthermore, hepatic FFA concentrations were 1.48-fold higher in the HF group compared to normal mice (*p* < 0.05). This increase was partially reversed by RAF or ISO alone, but the most pronounced reduction (26.5%) was observed with combination therapy. Nuclear factor erythroid 2–related factor 2 (Nrf-2), a central regulator of antioxidant gene expression [33], was significantly downregulated in HF-fed mice. However, co-treatment with RAF and ISO markedly enhanced hepatic Nrf-2 levels (*p* < 0.05), indicating reactivation of endogenous antioxidant pathways. Altogether, these results underscore that the synergistic effects of RAF and ISO offer substantial protection against HF-induced oxidative liver stress, likely through reinforcement of enzymatic antioxidant systems and suppression of lipid peroxidation.

### 3.6. RAF Potentiated ISO’s Protective Effects Against Fructose-Induced Hepatic Injury

**Figure 6:**
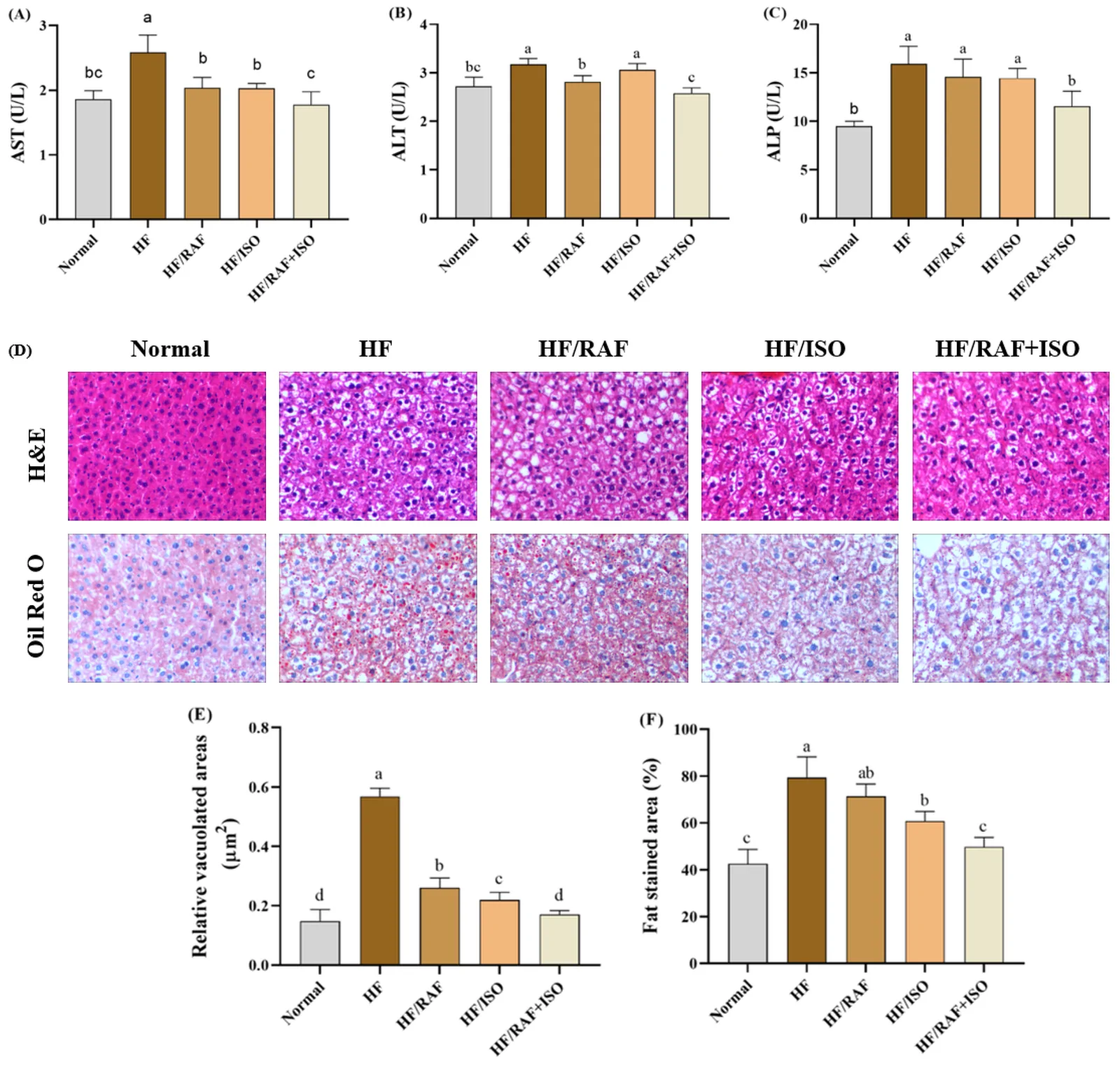
Effects of raffinose (RAF) and/or soybean isoflavones (ISO) on hepatic injury in high-fructose (HF)-fed mice. Serum aspartate aminotransferase (AST) (**A**), alanine aminotransferase (ALT) (**B**), and alkaline phosphatase (ALP) (**C**) activities; representative hematoxylin and eosin (H&E)-stained liver sections and relative vacuolated areas in liver tissues (**D**); Oil Red O staining (**E**); and lipid-stained areas (**F**). Values are expressed as the mean ± SD (*n* = 8). Scale bar = 100 μm. Different letters indicate significant differences among groups (*p* < 0.05).

Increased activities of liver enzymes such as AST, ALT, and ALP are established indicators of hepatocellular damage, often reflecting oxidative injury to cell membranes and mitochondria [34]. As presented in Figure 6A–C, HF-fed mice exhibited significantly elevated serum levels of these enzymes compared to the control group (*p* < 0.05). While monotherapy with either RAF or ISO effectively reduced AST activity, neither was sufficient to lower ALT or ALP. In contrast, co-administration of RAF and ISO significantly decreased all three enzyme levels (*p* < 0.05), indicating a more comprehensive hepatoprotective effect when the two agents were combined.

Histological evaluation further supported these findings. In H&E-stained liver sections, the normal group displayed intact hepatic architecture without signs of edema or cellular degeneration. Conversely, prolonged HF intake for 10 weeks led to pronounced liver injury characterized by cell shrinkage, membrane blurring, cytoplasmic condensation, and vacuolization (Figure 6D,E), consistent with the “first hit” mechanism of NAFLD progression [18,35]. RAF or ISO treatment alone moderately alleviated these histopathological changes, while their combined application resulted in more substantial restoration of normal liver morphology (*p* < 0.05).

Moreover, Oil Red O staining revealed substantial hepatic lipid accumulation in the HF group, with the stained fat area nearly doubled compared to the normal group (*p* < 0.05, Figure 6F). Monotherapy with either RAF or ISO led to gradual improvements, but co-treatment most effectively reduced intracellular lipid deposition, yielding sparse and evenly distributed lipid droplets that resembled those in healthy tissue (*p* < 0.05, Figure 6D,F). Collectively, these data suggest that RAF significantly amplifies ISO’s hepatoprotective efficacy against fructose-induced steatosis through synergistic actions on both biochemical and structural levels.

### 3.7. RAF + ISO Co-Treatment Mitigated Hepatic Inflammatory Responses in HF-Fed Mice

**Figure 7:**
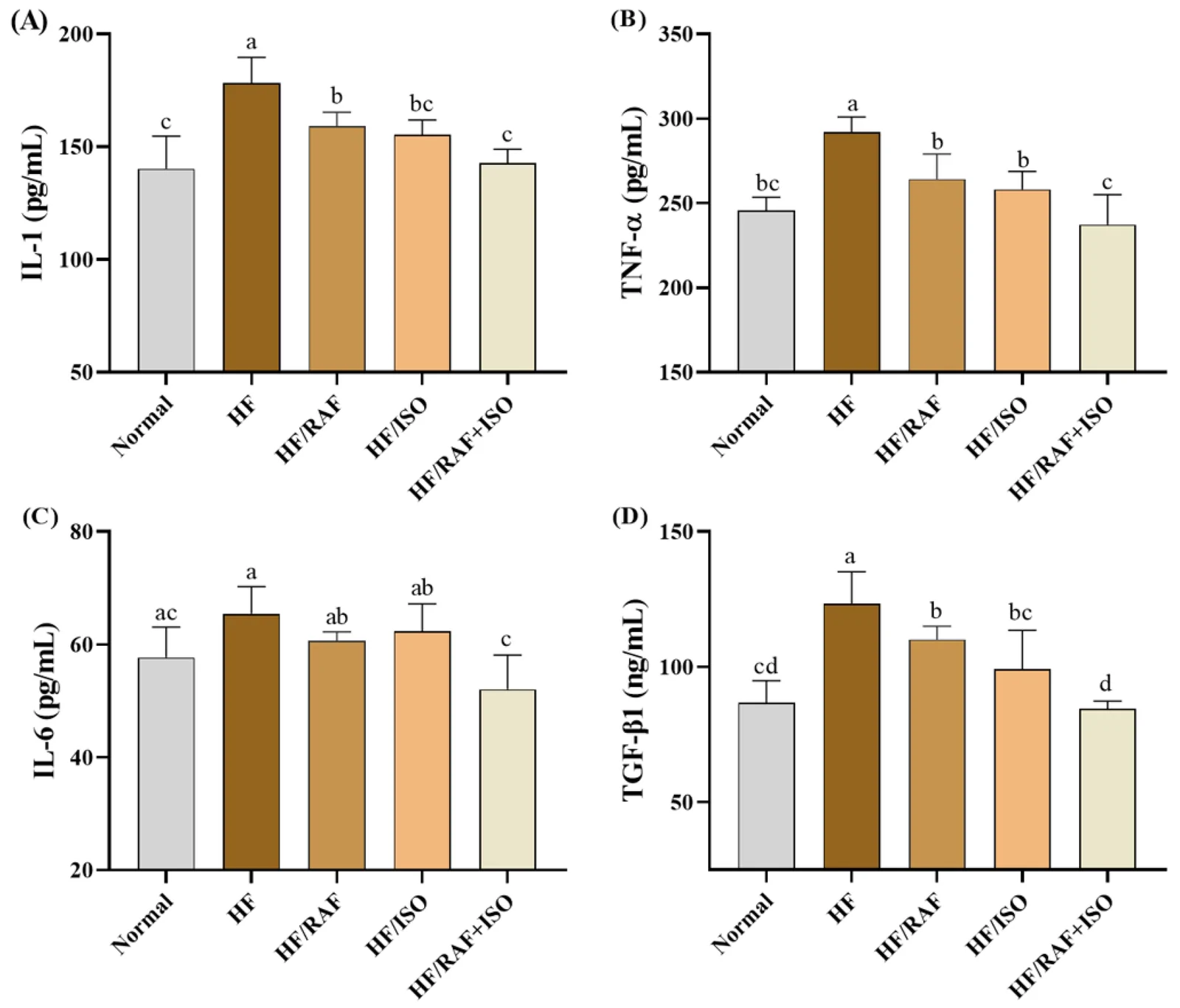
Hepatic levels of interleukin-1 (IL-1) (**A**), tumor necrosis factor-α (TNF-α) (**B**), interleukin-6 (IL-6) (**C**), and transforming growth factor-β1 (TGF-β1) (**D**) in the different experimental groups. HF, high fructose; RAF, raffinose; ISO, soybean isoflavones. Values are expressed as the mean ± SD (*n* = 8). Different letters indicate significant differences among groups (*p* < 0.05).

Oxidative damage and lipid peroxidation are known to induce pro-inflammatory cytokines, forming a critical component of the “second hit” mechanism in the pathogenesis of nonalcoholic liver disease [16,30,31]. As shown in Figure 7A,B, hepatic levels of IL-1 and TNF-α were significantly elevated—by 27.0% and 18.8%, respectively—in HF-fed mice compared to controls (*p* < 0.05). Both RAF and ISO treatments independently attenuated these elevations, whereas the combined treatment resulted in a more pronounced reduction of IL-1 by 24.9% and TNF-α by 23.1% (*p* < 0.05).

Although IL-6 levels showed a slight upward trend following HF exposure, this increase did not reach statistical significance (*p* > 0.05, Figure 7C). Nonetheless, the RAF + ISO combination significantly decreased IL-6 expression compared to the HF group (*p* < 0.05), achieving a greater inhibitory effect than either compound alone. Additionally, hepatic fibrosis associated with insulin resistance has been linked to increased expression of transforming growth factor-beta 1 (TGF-β1), a key profibrotic mediator that promotes extracellular matrix deposition and collagen synthesis [36,37]. In our study, HF-fed mice showed a notable rise in hepatic TGF-β1, which was significantly suppressed by both RAF and ISO treatments (*p* < 0.05, Figure 7D). Notably, the greatest reduction was observed in the RAF + ISO group, further supporting their synergistic anti-inflammatory and anti-fibrotic potential. In summary, the observed decreases in hepatic IL-1, TNF-α, IL-6, and TGF-β1 levels following combination therapy suggest that RAF may potentiate ISO’s protective effects by enhancing its intestinal absorption, thereby reinforcing its immunoregulatory impact under HF-induced hepatic stress.

## 4. Conclusions

This study provides experimental evidence that raffinose (RAF) can enhance the intestinal absorption of soybean isoflavones (ISO). The combined administration of RAF and ISO exerted greater protective effects against high-fructose-induced metabolic disturbances and hepatic injury than either component alone, as evidenced by improvements in glucose and lipid metabolism, antioxidant defense, adipogenesis-related factors, and inflammatory responses. These beneficial effects may reflect not only the enhanced bioavailability of ISO but also the complementary metabolic effects of RAF itself. From a practical perspective, the combination of RAF, a nondigestible oligosaccharide, and ISO, a soybean-derived bioactive component, represents a promising strategy for the development of multi-component functional foods designed to support metabolic and liver health. Further studies, particularly clinical investigations, are warranted to validate these effects in humans and facilitate their translation into functional food applications.

## Figures and Tables

**Table 1:** Effects of RAF, ISO and their combination (RAF + ISO) on diet consumption, body weight, liver weight, hepatosomatic index, fat weight and fat index in mice fed with HF water for 10 weeks.

	Normal	HF	HF/RAF	HF/ISO	HF/RAF + ISO
Food intake (g/mouse per day)	4.13 ± 0.43 ^a^	2.17 ± 0.41 ^b^	2.05 ± 0.37 ^b^	2.33 ± 0.33 ^b^	2.28 ± 0.35 ^b^
Water consumption (mL/mouse per day)	7.88 ± 0.58 ^a^	7.84 ± 0.76 ^a^	7.68 ± 0.51 ^a^	7.65 ± 0.48 ^a^	7.70 ± 0.56 ^a^
Final body weight (g)	41.9 ± 2.22 ^b^	46.5 ± 2.93 ^a^	41.5 ± 2.81 ^b^	40.5 ± 0.54 ^b^	40.3 ± 0.62 ^b^
Body weight gain (g)	9.51 ± 0.51 ^b^	12.1 ± 0.43 ^a^	8.17 ± 0.51 ^d^	7.76 ± 0.59 ^c^	7.17 ± 0.42 ^c^
Liver weight (g)	1.40 ± 0.14 ^d^	2.33 ± 0.12 ^a^	1.88 ± 0.30 ^bc^	2.06 ± 0.17 ^b^	1.70 ± 0.12 ^c^
Hepatosomatic index	3.34 ± 0.06 ^d^	5.01 ± 0.03 ^a^	4.53 ± 0.05 ^b^	5.08 ± 0.05 ^a^	4.22 ± 0.07 ^c^
Abdominal fat weight (g)	1.47 ± 0.25 ^e^	2.41 ± 0.32 ^a^	1.92 ± 0.29 ^b^	2.08 ± 0.30 ^c^	1.80 ± 0.23 ^d^
Abdominal fat index	3.51 ± 0.11 ^d^	5.18 ± 0.10 ^a^	4.63 ± 0.10 ^b^	5.13 ± 0.09 ^b^	4.46 ± 0.06 ^c^

Note: Values are expressed as the mean ± SD of 8 mice in each group. Different letters above numbers indicate significant differences in the mean of the same sample (*p* < 0.05).

## Data Availability

The original contributions presented in this study are included in the article. Further inquiries can be directed to the corresponding author.
